# Supplementary material for: Structural mechanism of human oncochannel TRPV6 inhibition by the natural phytoestrogen genistein
Source: Nat Commun. 2023 May 9;14:2659. doi: 10.1038/s41467-023-38352-5 (PMC10169861; doi:10.1038/s41467-023-38352-5)
Supplement: Supplementary file 1 — Supplementary Information [file 41467_2023_38352_MOESM1_ESM.pdf]

Supplementary Information

Structural mechanism of human oncochannel TRPV6 inhibition by the natural phytoestrogen genistein

Arthur Neuberger[1], Yury A. Trofimov[2], Maria V. Yelshanskaya[1], Kirill D. Nadezhdin[1], Nikolay A. Krylov[2], Roman G. Efremov[2], Alexander I. Sobolevsky[1,*]

[1] Department of Biochemistry and Molecular Biophysics, Columbia University, New York, NY, USA

[2] Shemyakin-Ovchinnikov Institute of Bioorganic Chemistry, Russian Academy of Sciences, Moscow, Russia

* Corresponding author. E-mail: **as4005@cumc.columbia.edu**

This PDF file includes:

Supplementary Figures 1-8

Supplementary Tables 1-2

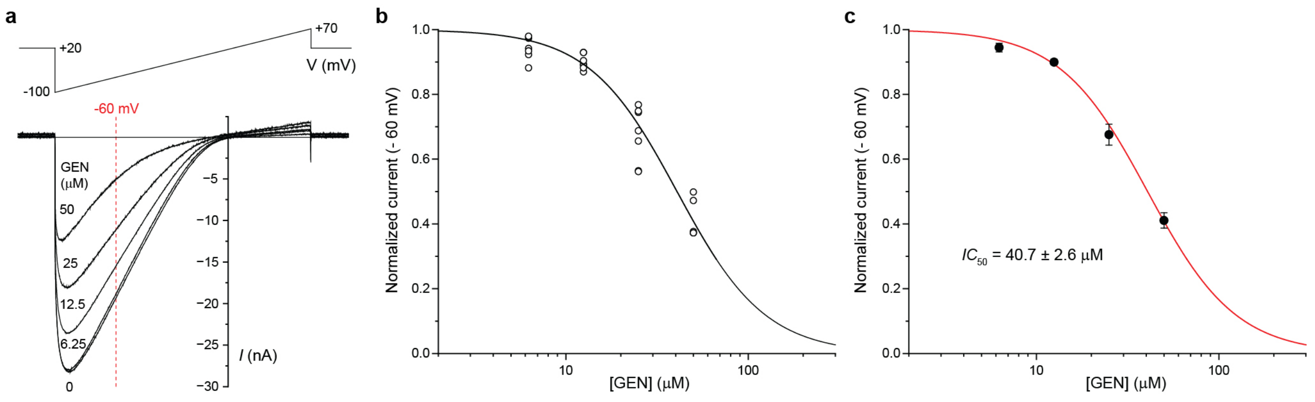

Supplementary Fig. 1 | Concentration dependence of TRPV6-mediated current inhibition by genistein. a Whole-cell patch-clamp currents recorded from HEK 293S cells expressing hTRPV6 in response to −100 to 70 mV voltage ramp at different concentrations of genistein (labeled, in μM). **b**,**c** Concentration dependence of genistein inhibition for individual (**b**) and average (**c**) measurements of TRPV6-mediated current amplitude at −60 mV, normalized to the value in the absence of genistein (0 μM). The curves show fitting of the average normalized current concentration dependence with logistic equation and IC_{50} = 40.7 ± 2.6 μM and n_{Hill} = 1.80 ± 0.13 (n = 7 independent experiments). Data are presented as mean values ± SEM.

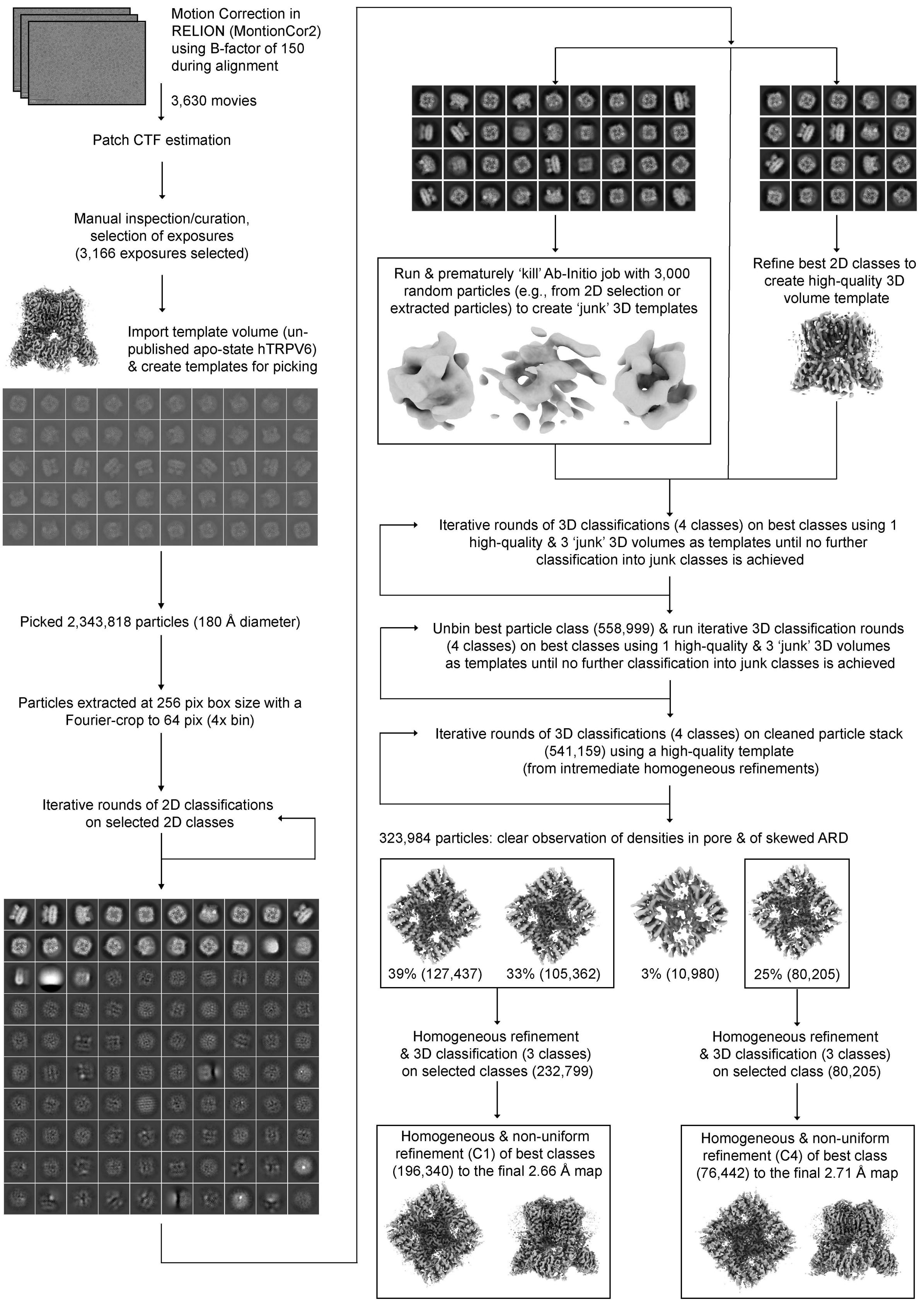

Supplementary Fig. 2 | 3D reconstruction workflow. 3D reconstruction workflow steps are illustrated by examples of micrographs, 2D class averages and cryo-EM densities.

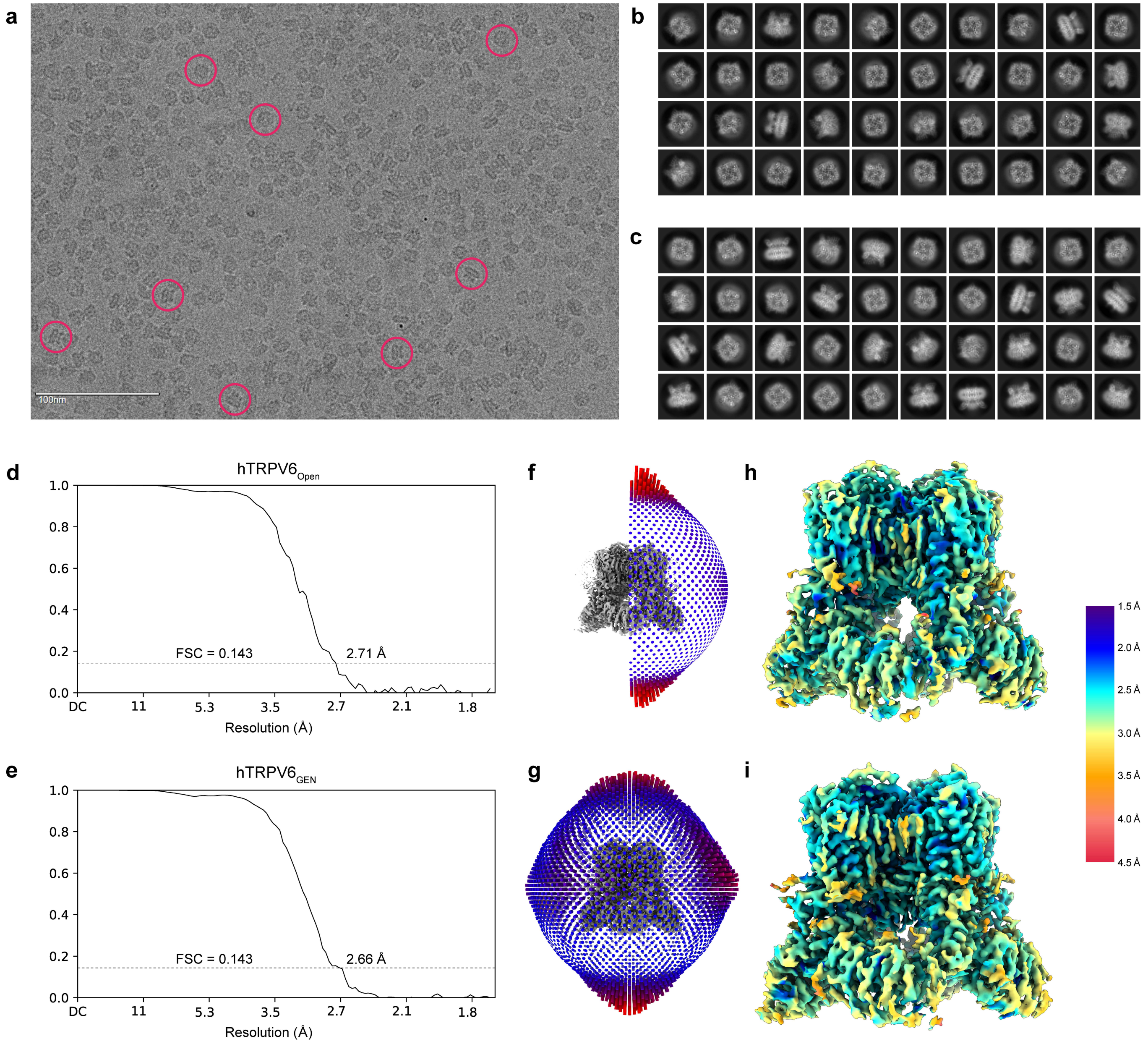

Supplementary Fig. 3 | Overview of cryo-EM data for hTRPV6 in the presence of genistein. **a** Representative micrograph with example particles circled in pink. A total of 3,630 such micrographs were collected, manually inspected and those with outliers in defocus values, ice thickness, and astigmatism as well as those with lower predicted CTF-correlated resolution (higher than 5 Å) were excluded (individually assessed for each parameter relative to the overall distribution), with 3,166 micrographs left for further processing (see Methods and Supplementary Fig. 2). **b**,**c** Representative 2D class averages for hTRPV6$_{Open}$ (**b**) and hTRPV6$_{GEN}$ (**c**). **d**,**e** FSC curves for hTRPV6$_{Open}$ (**d**) and hTRPV6$_{GEN}$ (**e**). **f**,**g** Euler angle distribution of particles contributing to final reconstructions of hTRPV6$_{Open}$ (**f**) and hTRPV6$_{GEN}$ (**g**) with larger red cylinders representing orientations comprising more particles. **h**,**i** Local resolution presented as coloring of the cryo-EM maps of hTRPV6$_{Open}$ (**h**) and hTRPV6$_{GEN}$ (**i**).

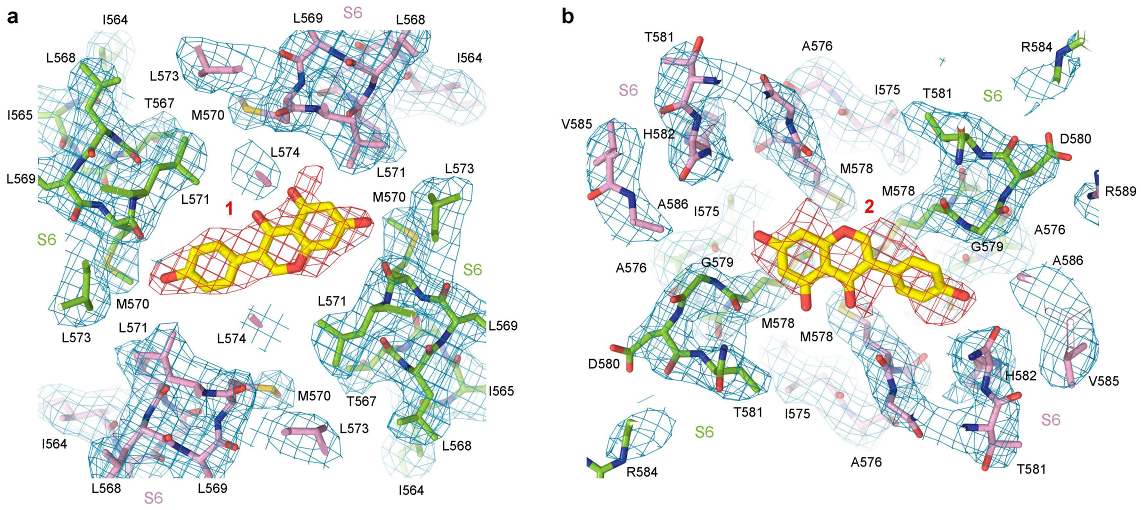

Supplementary Fig. 4 | Density for genistein molecules and surrounding protein. a,b Sites 1 (**a**) and 2 (**b**) of genistein viewed intracellularly along the channel pore, with molecules of genistein (yellow) and TRPV6 subunits A and C (green) as well as B and D (pink) shown in sticks and density for genistein and the surrounding protein as red and blue mesh, respectively.

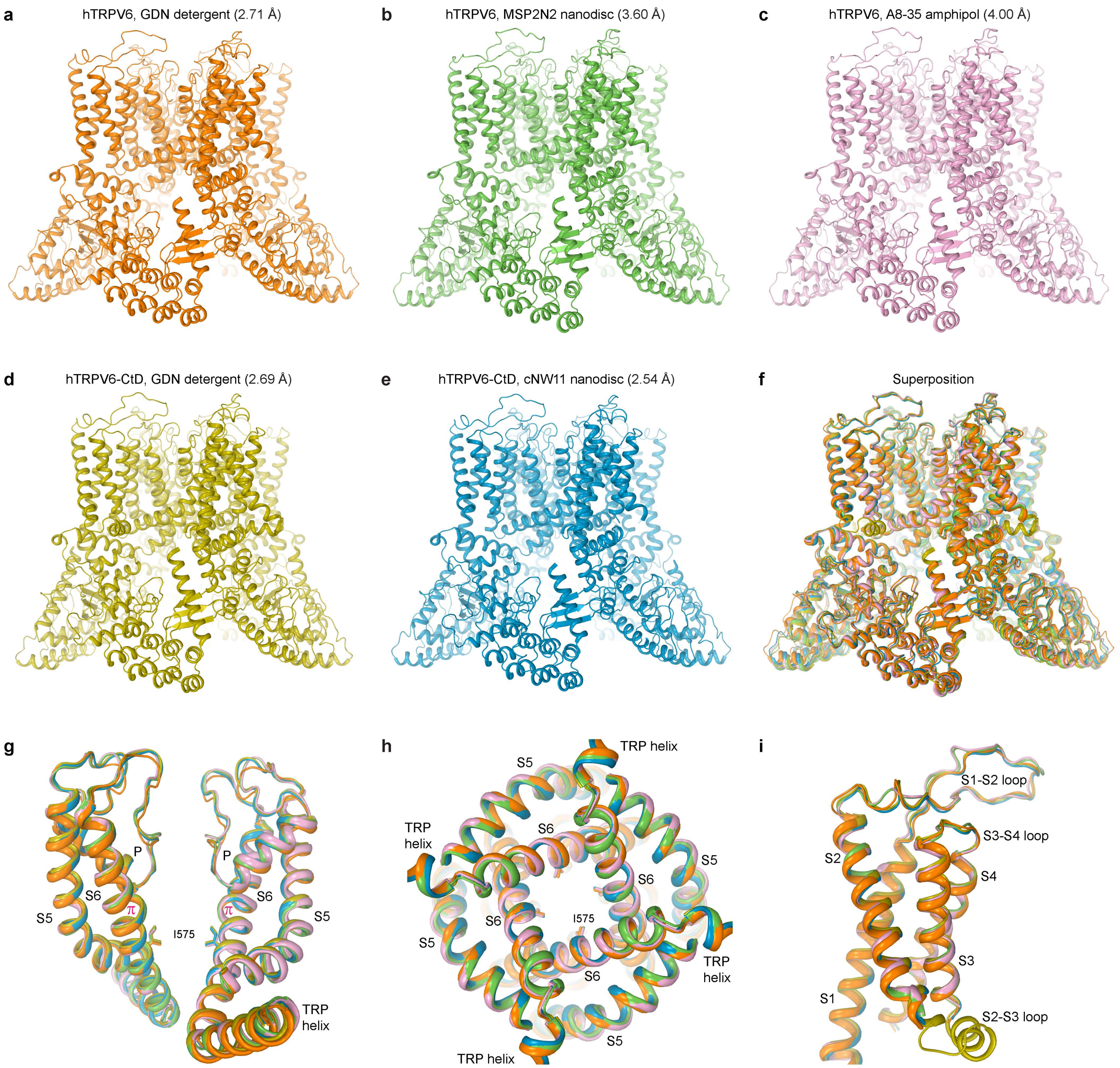

Supplementary Fig. 5 | Comparison of human TRPV6 open-state structures. a-e Open-state (apo) cryo-EM structures of full-length hTRPV6 viewed parallel to membrane in GDN detergent (**a**, orange), MSP2N2 nanodisc (**b**, green, PDB ID: 6BO8) and A8-35 amphipol (**c**, pink, PDB ID: 6BO9), as well as hTRPV6-CtD in GDN detergent (**d**, olive) and cNW11 nanodisc (**e**, blue). **f** Superposition of structures shown in **a**-**e**. **g**,**h** Parallel to membrane (**g**) and intracellular (**h**) close-up views of the pore-forming domains in structures superposed in **f**. Only two of four subunits are shown in **g**, with the front and back subunits omitted for clarity. **i** Parallel to membrane close-up view of S1-S4 in structures superposed in **f**.

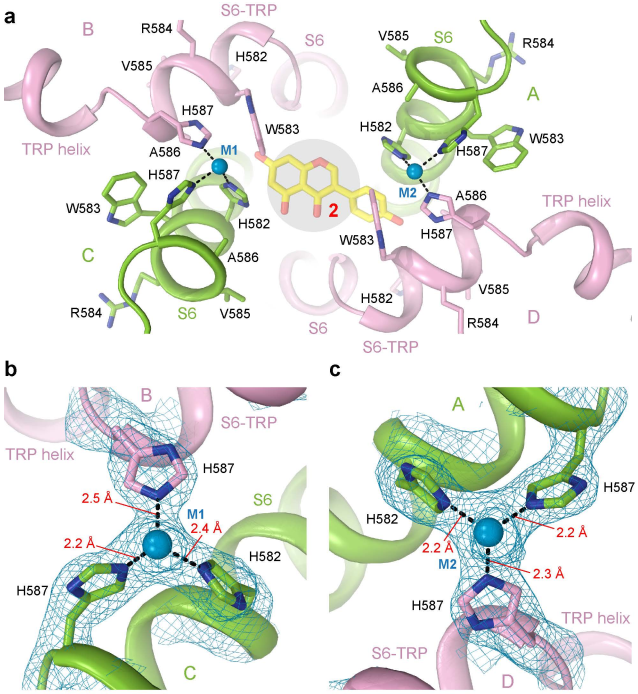

Supplementary Fig. 6 | Cryo-EM density for the metal ions bound at the TRPV6$_{GEN}$ pore intracellular entrance. a View of the intracellular entrance to the pore (grey circle in the middle), with subunits A and C colored green, B and D pink, and metal ions (M1 and M2) shown as blue spheres. The molecule of genistein at site 2 and residues surrounding and contributing to the metal binding sites are shown in sticks. **b**,**c** Close-up views of M1 (**b**) and M2 (**c**) coordinated by H582 and H587, with cryo-EM density for the histidines and metal ions shown as blue mesh and distances between them indicated (red).

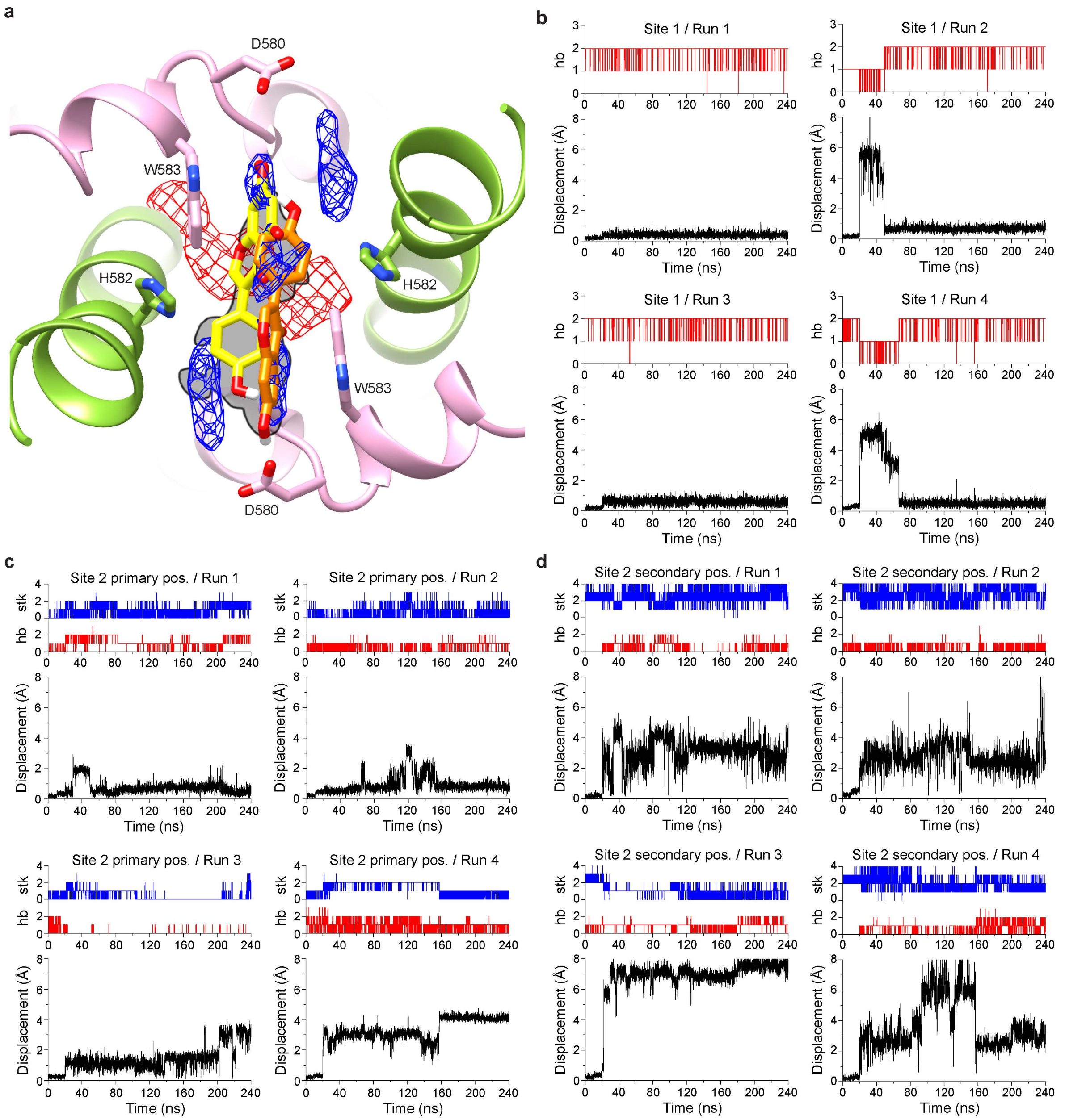

Supplementary Fig. 7 | MD simulations of genistein binding to sites 1 and 2. a Intracellular view of genistein at the secondary position in site 2. Gray filling shows the density for genistein heavy atoms averaged over the MD runs. The non-protein cryo-EM densities for site 2 are shown as red and blue mesh (associated with genistein molecules at the primary and secondary positions, respectively). Protein subunits A/C (green) and B/D (pink) are shown as cartoon models, with residues forming hydrogen bonds and π-stacking interactions with genistein shown in sticks and labeled. The most populated MD states of genistein at the secondary position in site 2 are illustrated by representative yellow and orange stick models. **b-d** Measurements for four individual MD runs are shown for genistein in site 1 (**b**), and at the primary (**c**) and secondary (**d**) positions in site 2. Black curves show the displacement of the center of genistein molecule (center of mass of the heavy atoms in the ring structures of the molecule) relative to its initial position in each MD run (genistein position is fixed during the first 20 ns). Red and blue curves show the number of genistein-protein hydrogen bonds and π-stacking interactions, respectively.

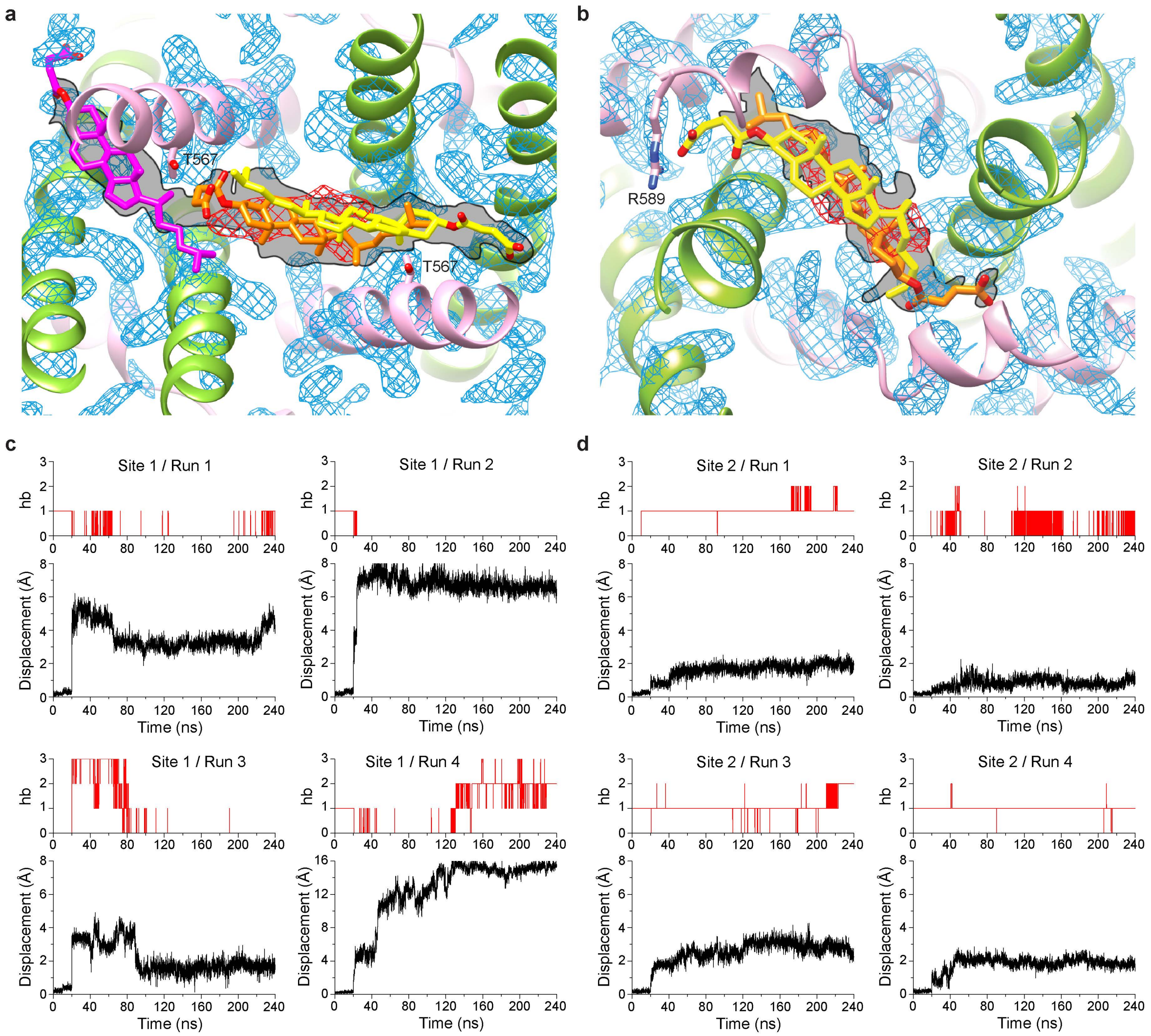

Supplementary Fig. 8 | MD simulations of CHS binding to sites 1 and 2. a,**b** Extracellular view of site 1 (**a**) and intracellular view of site 2 (**b**). Gray filling shows the density for CHS heavy atoms averaged over the MD runs. The experimental cryo-EM protein and non-protein densities are shown as blue and red mesh, respectively. Protein subunits A/C (green) and B/D (pink) are shown as cartoon models, with residues forming hydrogen bonds with CHS shown in sticks and labeled. The populated MD states of CHS are illustrated by representative yellow, orange, and magenta stick models. **c**,**d** Measurements for four individual MD runs are shown for sites 1 (**c**) and 2 (**d**). Black curves show the displacement of the center of CHS molecule (center of mass of the heavy atoms in the ring structures of the molecule) relative to its initial position in each MD run (CHS position is fixed during the first 20 ns). Red curves show the number of CHS-protein hydrogen bonds.

Supplementary Table 1 | Cryo-EM data collection, refinement, and validation statistics

**Structure**	**hTRPV6$_{GEN}$**	**hTRPV6$_{Open}$**
EMDB accession code	EMD-29343	EMD-29344
PDB accession code	8FOA	8FOB
**Data collection and processing**		
Magnification	130,000x	130,000x
Voltage (kV)	300	300
Electron exposure ($e^{-}Å^{-2}$)	60	60
Defocus range (μm)	−0.8 to −2.0	−0.8 to −2.0
Reported pixel size (Å)	0.84	0.84
Exposures (no.)	3,630	3,630
**Processing software**		
Motion correction	RELION 4.0 (MotionCor2)	RELION 4.0 (MotionCor2)
CTF estimation	cryoSPARC v3.3	cryoSPARC v3.3
Platform software for particle picking	cryoSPARC v3.3	cryoSPARC v3.3
Software for 2D/3D class. & refinements	cryoSPARC v3.3	cryoSPARC v3.3
Symmetry imposed	C1	C4
Initial particle images (no.)	2,343,818	2,343,818
Final particle images (no.)	196,340	76,442
Map resolution (Å) FSC 0.143	2.66	2.71
**Refinement**		
Initial models used (PDB code)	7S89	7S89
Model resolution (Å)	2.66	2.71
FSC threshold	0.143	0.143
Map sharpening *B* factor ($Å^2$)	−100.4	−101.4
**Model composition**		
Non-hydrogen atoms	21,709	21,202
Protein residues	2,460	2,392
Ligands	35	34
Water	38	52
B factors ($Å^2$)		
Protein	73.00	65.67
Ligands	20.44	34.29
Water	13.20	16.45
R.m.s. deviations		
Bond lengths (Å)	0.005	0.007
Bond angles (°)	0.953	1.005
**Validation**		
MolProbity score	1.44	1.80
Clash score, all atoms	1.64	3.41
Poor rotamers (%)	0.00	0.00
Ramachandran plot		
Favored (%)	93.98	89.66
Allowed (%)	6.02	10.00
Disallowed (%)	0.00	0.34

Supplementary Table 2 | List of MD runs

Run name	Ligand starting position	Description
gen_site1_run1	pose 1 (like in the cryo-EM model)	Differently oriented genistein molecules in site 1*
gen_site1_run2	pose 2	
gen_site1_run3	pose 3	
gen_site1_run4	pose 4	
gen_site2_run1	pose 1 (like in the cryo-EM model)	Differently orientated two genistein molecules at the primary and secondary positions in site 2**
gen_site2_run2	pose 2	
gen_site2_run3	pose 3	
gen_site2_run4	pose 4	
chs_run1	pose 1	Differently orientated two CHS molecules in sites 1 and 2
chs_run2	pose 2	
chs_run3	pose 3	
chs_run4	pose 4	

* In runs *gen_site1_run1-4*, the second genistein molecule was inserted into the primary position of site 2, but it was unstable in MD without the ligand in the secondary position.

** In runs *gen_site2_run1-4*, genistein was also inserted into site 1, but it was unstable in MD because of more extended constrictions applied to the protein (the distances were constrained between each residue in the region 563-578 for *gen_site1_run1-4* and in the region 536-586 for *gen_site2_run1-4*). These constrictions prevented protein adaptation for the ligand binding in site 1.
