# Supplementary material for: Structural mechanism of human oncochannel TRPV6 inhibition by the natural phytoestrogen genistein
Source: Nat Commun. 2023 May 9;14:2659. doi: 10.1038/s41467-023-38352-5 (PMC10169861; doi:10.1038/s41467-023-38352-5)
Supplement: Supplementary file 3 — Description of additional supplementary files [file 41467_2023_38352_MOESM3_ESM.pdf]

Description of additional supplementary files

Supplementary Movie 1:

This movie shows transformation of human TRPV6 from the open state (orange) to the closed blocked state (blue) and back upon binding and dissociation of the inhibitor genistein (yellow), respectively. The movie was made as a morph between the TRPV6Open and TRPV6GEN structures.

Supplementary Data Set 1:

The data set includes supplementary pdb files of atomic coordinates for the transmembrane domain of human TRPV6 (residues 454-589) and ligands (Genistein or CHS) with the time step of 20 ns obtained in MD simulations (including the initial configuration at t = 0). The pdb file names correspond to the names of MD runs described in Supplementary Table 2 (also included as a Word file into the MD supplementary data set).
