# Supplementary material for: Structural mechanism of human oncochannel TRPV6 inhibition by the natural phytoestrogen genistein
Source: Nat Commun. 2023 May 9;14:2659. doi: 10.1038/s41467-023-38352-5 (PMC10169861; doi:10.1038/s41467-023-38352-5)
Supplement: Supplementary file 4 — Supplementary Data 1 [file 41467_2023_38352_MOESM4_ESM.zip › MD states/List of MD runs.docx]

List of MD runs

| **Run name** | **Ligand starting position** | **Description** |
| --- | --- | --- |
| gen_site1_run1 | pose 1 (like in cryo-EM model) | Differently oriented genistein molecules in site 1* |
| gen_site1_run2 | pose 2 |  |
| gen_site1_run3 | pose 3 |  |
| gen_site1_run4 | pose 4 |  |
| gen_site2_run1 | pose 1 (like in cryo-EM model) | Differently orientated two genistein molecules at the primary and secondary positions in site 2** |
| gen_site2_run2 | pose 2 |  |
| gen_site2_run3 | pose 3 |  |
| gen_site2_run4 | pose 4 |  |
| chs_run1 | pose 1 | Differently orientated two CHS molecules in sites 1 and 2 |
| chs_run2 | pose 2 |  |
| chs_run3 | pose 3 |  |
| chs_run4 | pose 4 |  |

* – in runs *gen_site1_run1-4* the second genistein molecule was inserted in the primary position of site 2, but it was unstable in MD without the ligand in the secondary position. ** – in runs *gen_site2_run1-4* genistein was also inserted in site 1, but it was unstable in MD because of more extended constrictions applied to the protein (the distances were constrained between each residue in the region 563-578 for *gen_site1_run1-4* and in the region 536-586 for *gen_site2_run1-4*). These constrictions prevented protein adaptation for the ligand binding in site 1.
